# Role of glucocorticoid and mineralocorticoid receptors in rainbow trout (*Oncorhynchus mykiss*) skeletal muscle: A transcriptomic perspective of cortisol action

**DOI:** 10.3389/fphys.2022.1048008

**Published:** 2023-01-06

**Authors:** Jorge E. Aedo, Rodrigo Zuloaga, Daniela Aravena-Canales, Alfredo Molina, Juan Antonio Valdés

**Affiliations:** ^1^ Departamento Ciencias Biológicas, Facultad de Ciencias de la Vida, Universidad Andres Bello, Santiago, Chile; ^2^ Interdisciplinary Center for Aquaculture Research (INCAR), Concepción, Chile

**Keywords:** cortisol, glucocorticoid receptor, mineralocorticoid receptor, RNA-seq, skeletal muscle

## Abstract

Cortisol is an essential regulator of neuroendocrine stress responses in teleost. Cortisol performs its effects through the modulation of glucocorticoid receptor (GR) and mineralocorticoid receptor (MR), activating gene expression. Until now the contribution of both receptors in the global transcriptional response in teleost skeletal muscle has not been explored. To understand in a comprehensive and global manner how GR and MR modulates the skeletal muscle transcriptomic response, we performed RNA-seq analysis. Juvenile rainbow trout (*Oncorhynchus mykiss*) pretreated with a suppressor of endogenous cortisol production were intraperitoneally injected with cortisol (10 mg/kg). We also included a treatment with mifepristone (GR antagonist) and eplerenone (MR antagonist) in the presence or absence of cortisol. cDNA libraries were constructed from the skeletal muscle of rainbow trout groups: vehicle, cortisol, mifepristone, eplerenone, mifepristone/cortisol and eplerenone/cortisol. RNA-seq analysis revealed that 135 transcripts were differentially expressed in cortisol vs. mifepristone/cortisol group, mainly associated to inflammatory response, ion transmembrane transport, and proteolysis. In the other hand, 68 transcripts were differentially expressed in cortisol vs. eplerenone/cortisol group, mainly associated to muscle contraction, and regulation of cell cycle. To validate these observations, we performed *in vitro* experiments using rainbow trout myotubes. In myotubes treated with cortisol, we found increased expression of *cxcr2*, *c3*, and *clca3p* mediated by GR, associated with inflammatory response, proteolysis, and ion transmembrane transport, respectively. Contrastingly, MR modulated the expression of *myh2* and *gadd45g* mainly associated with muscle contraction and regulation of cell cycle, respectively. These results suggest that GR and MR have a differential participation in the physiological response to stress in teleost skeletal muscle.

## 1 Introduction

Although the concept of stress has a bad reputation, physiological stress response helps to promote survival of an organism during threating situations (Balasch and Tort, 2019). The biochemical reactions that complements stress response plays an important role in the metabolic adjustments that are critical for meeting the increased energy requirement (Ellis et al., 2012). The stress response in teleost is a biphasic process. It starts with an initial release of catecholamines from the kidney chromaffin tissue, producing a short latency increase in plasma catecholamine levels (Suarez-Bregua et al., 2018). This is followed by the secretion of cortisol from the interrenal tissue, generating a long-latency and extended release of cortisol to the bloodstream (Wendelaar Bonga, 1997). Cortisol-mediated response in teleost plays a fundamental role in early metabolic adaptation, in which the increased secretion of cortisol allows long-term glucose maintenance through the enhancement of gluconeogenesis and amino acid catabolism (Mommsen et al., 1999).

Cortisol binds and activates specific corticoid receptors in target tissues. There are two types of corticoid steroid receptors in fish: mineralocorticoid receptor (MR) and glucocorticoid receptor (GR) (Greenwood et al., 2003). These receptors (MR and GR) evolved from an ancestral corticoid receptor (CR) in jawless fish through gene duplication and divergence (Baker and Katsu, 2019). The appearance of the MR in evolution occurred before the appearance of aldosterone synthase (Baker, 2003). Therefore, aldosterone, the main physiological mineralocorticoid in mammals, is not present in teleost fish (Fuller et al., 1976). MRs have a high affinity for cortisol, while GRs have a low affinity (Gilmour, 2005). Most fish possess one MR and two GR isoforms, GR1 and GR2 (Baker et al., 2013). In the rainbow trout, GR2 is more sensitive to reduced plasma cortisol levels, whereas GR1 is more sensitive to higher levels, a condition caused by acute stressors (Bury and Sturm, 2007). Once inside the cell, cortisol binds to cytoplasmic GR or MR and induces induce their dissociation from molecular chaperones. The hormone–receptor complex then translocate to the nucleus, where it dimerizes and binds to corticosteroid response elements of target genes (Aluru and Vijayan, 2009). Depending on the co-factors recruited, this binding leads to trans-activation or trans-repression of these genes. This mode of action is known as the genomic pathway because it involves gene expression (Bury and Sturm, 2007). In addition to its genomic pathway, cortisol also has effects characterized by rapid second messenger activation, which leads to acute cellular responses (Borski et al., 2001). This mode of action is known as the non-genomic pathway.

Although there are several studies that analyze the molecular response to cortisol in skeletal muscle, the contribution of GR and MR in these processes is unknown. Recently, it was determined that exogenous cortisol treatment is capable of emulating an acute stress condition in rainbow trout, promoting the differentially expression of transcripts associated to mRNA processing, protein ubiquitination, and transcription regulation (Aedo et al., 2019c). Additionally, it was determined the non-genomic action of cortisol associated with a membrane receptor complementary to the genomic actions (Aedo et al., 2019a). In the present work, we performed RNA-seq analysis to evaluate the early transcriptomic response of the skeletal muscle in juvenile rainbow trout treated with cortisol in the presence or absence of mifepristone or eplerenone. The use of mifepristone and eplerenone, both GR and MR antagonists, allowed us to discriminate between the participation of these receptors in cortisol-induced gene expression in rainbow trout skeletal muscle. Thus, we determined, for the first time, potential target genes, biological processes and signaling pathways modulated through these receptors.

## 2 Methodology

### 2.1 Ethics statement

The study adhered to animal welfare procedures and was approved by the bioethical committees of the Universidad Andres Bello and the National Commission for Scientific and Technological Research of the Chilean government.

### 2.2 Protocol experiment

Juvenile rainbow trout (9.70 g ± 26) were obtained from Pisciculture Rio Blanco (V region, Chile). Fish were maintained under natural temperatures and light:dark photoperiod conditions (14°C ± 1°C and L:D 12:12) and fed daily with commercial pellets, except for the day before the *in vivo* protocol. All fish were anesthetized with benzocaine in water (25 mg/L) and intraperitoneally treated with metyrapone (Sigma-Aldrich, St. Luis, MO, USA) (1 mg/kg of fish) for 1 h and then randomly divided into six groups. A group without treatment was maintained to quantify basal cortisol levels. Fish in the first and second groups were treated with vehicle solution (DMSO, PBS 1X) and cortisol (Sigma-Aldrich, St. Luis, MO, USA) at the concentration of 10 mg/kg. Fish in the third and fourth groups were treated with mifepristone (1 mg/kg) and mifepristone (1 mg/kg) plus cortisol (10 mg/kg). Finally, fish in the fifth and sixth groups were treated with eplerenone (1 mg/kg) and eplerenone (1 mg/kg) plus cortisol (10 mg/kg). After 3 hours of incubation with each compound, all the fish (*n* = 24) were euthanized by overdoses of benzocaine (300 mg/L). Blood was collected from the caudal vessel with a 1 mL heparinized (10 mg/mL) syringe. Plasma was obtained by centrifugation at 5,000 *g* for 10 min, immediately frozen in liquid nitrogen and stored at −80°C until analysis. Myotomal skeletal muscle was obtained from the epaxial area for all of the sampled fish, triturated after freezing with liquid nitrogen, placed in RNA later (Sigma-Aldrich, Sant Louis, MO, USA) for 24 h at 4°C and then stored at −80°C for subsequent analysis. Plasma cortisol and glucose levels were measured using the Cayman cortisol (Cayman Chemical, Ann Arbor, MI, USA) and glucose kits (Abcam, Cambridge, United Kingdom), respectively.

### 2.3 Library construction and sequencing

RNA was extracted from the skeletal muscle of the vehicle, cortisol, mifepristone, mifepristone/cortisol, eplerenone, and eplerenone/cortisol treated fish using the RNeasy Mini Kit (Qiagen, TX, United States) following the manufacturer’s recommendations. RNA integrity was confirmed by electrophoresis using a 1.2% agarose gel and the capillary electrophoresis Fragment Analyzer Automated CE System (Advanced Analytical Technologies, Inc., IA, United States). Only RNA samples with RQN ≥8.5 were used in further analyses. The amount of total RNA was measured by a fluorometer using the Qubit RNA BR assay kit (Invitrogen, CA, United States). Twenty-four cDNA libraries corresponding to 1 µg of an RNA of each condition, were generated using the TruSeq RNA Sample Preparation kit v2 (Illumina, United States). A total of 24 libraries were sequenced using a paired-end strategy (2 bp × 100 bp) with the HiSeq 2,500 (Illumina) platform of Macrogen (Seul, Korea). The raw data were deposited into the Sequence Read Archive (SRA) available on the NCBI database (PRJNA518130).

### 2.4 Raw data processing and RNA-seq analysis

Raw reads were analyzed using CLC genomic workbench 9.0 software (CLC bio—Qiagen, TX, United States). Adapters were removed, and raw data were trimmed with low quality reads (Q < 20) and reads length >50 bp. Trimmed reads were mapped onto the reference rainbow trout genome using default mapping parameters: mismatches = 2, minimum fraction length = .9, minimum fraction similarity = .8, and maximum hits per read = 5.

Differential expression analysis *in silico* was based on reads that uniquely mapped to the reference and the proportional-based statistical K-test. Transcripts with absolute fold-change values ≥ 2.0 and an FDR corrected *p*-value *<* .05 were considered differentially expressed transcripts (DETs). A comparison between the vehicle vs. cortisol groups considered potential genes regulated by the whole spectrum of cortisol action. Comparison between cortisol vs. mifepristone/cortisol and cortisol vs. eplerenone/cortisol were considered to identify potential genes regulated by cortisol and mediated by the glucocorticoid and mineralocorticoid receptors, respectively. In addition, comparison between vehicle vs. mifepristone and vehicle vs. eplerenone were included to identify potential genes regulated by glucocorticoid and mineralocorticoid receptors under endogenous suppressed cortisol production, respectively.

### 2.5 Functional annotation analysis

The DETs were examined against the DAVID resource (https://david.ncifcrf.gov/) and then categorized based on GO terms for molecular functions, biological processes, cellular components, and KEGG pathways. The gene ID of DETs were extracted and used as input to the DAVID GO enrichment analysis. To obtain the GO ID of each rainbow trout transcript, we performed a BLASTx search against different fish databases, including rainbow trout, Atlantic salmon (*Salmo salar*), Coho salmon (*Oncorhynchus kisutch*), zebrafish (*Danio rerio*) and Atlantic cod (*Gadus morhua*). Custom IDs set were selected for DAVID analysis as the “Background” Standard settings for ease (1) and gene count (2).

### 2.6 Rainbow trout myotube culture and cortisol treatments

Rainbow trout (*Oncorhynchus mykiss*) myotubes were prepared as previously reported (Espinoza et al., 2017). Briefly, skeletal muscle was obtained under sterile conditions and collected in Dulbecco’s Modified Eagle’s Medium (DMEM). After mechanical dissociation, the tissue was digested with a .2% solution of collagenase in DMEM for 1 h at 18°C. The suspension was centrifuged at 300 × g at 15°C for 5 min, and the resulting pellet was subjected to two series of enzymatic digestion with a .1% trypsin solution in DMEM for 20 min at 18°C. The cellular suspension was filtered through 40 μm nylon filters and seeded at a density of 2 × 10^6^ cells per well in plates that were previously treated with poly-L-lysine and laminin. Myoblast were incubated at 15°C for 7 days under an air atmosphere and in a proliferating medium (DMEM, 9 mm NaHCO3, 20 mm HEPES, 10% fetal bovine serum, 100 U/L penicillin and 10 mg/mL streptomycin). Myoblast were incubated for an additional 7 days differentiating medium (DMEM, 9 mm NaHCO3, 20 mm HEPES, 100 U/mL penicillin and 10 mg/mL streptomycin). Then, myotubes were preincubated for 30 min with or without the GR antagonist mifepristone (50 nM) or MR antagonist eplerenone (50 nM) (Sigma, St. Luis, MO) and stimulated with cortisol (500 nM) or vehicle solution (DMSO- PBS 1X) for 1, 3, and 6 h. After each treatment, cells were collected for RNA isolation. All treatments were performed with an *n* = 4, in two independent experiments.

### 2.6 Real-time PCR validation

RNA from rainbow trout myotube were extracted using Trizol (Invitrogen, United States), according to the manufacturer’s instructions. RNA was quantified using Nanodrop equipment. A total of 1 μg of RNA from skeletal muscle was reverse transcribed into cDNA using the QuantiTect reverse transcription kit (Qiagen, TX, United States). Primers to amplify the candidate genes evaluated in this study were designed using Primer 3 software (http://frodo.wi.mit.edu/primer3/) and validated with NetPrimer (http://www.premierbiosoft.com/netprimer/). Quantitative PCR was performed using a Stratagene MX3000P qPCR system (Stratagene, La Jolla, CA, United States). Each qPCR reaction mixture contained 7.5 μL of 2× Brilliant^®^ II SYBR^®^ master mix (Stratagene, La Jolla, CA, USA), 6 µL of cDNA (40-fold diluted) and 250 nM of each primer in 20 µL of final volume. Control reactions included a no template control and a control without reverse transcriptase. The list of primers used in this study are found in Supplementary Table S1. Amplifications were performed in triplicate with the following thermal cycling conditions: initial activation at 95°C for 2 min, followed by 40 cycles of 30 s of denaturation at 95°C, 30 s of annealing at 54°C–60°C and 30 s of elongation at 72°C. The presence of a single PCR product was confirmed by performing a melting curve analysis of the PCR products. Relative expression analysis was conducted using geNorm software (https://genorm.cmgg.be/), and results were expressed as the fold induction compared with the vehicle group and using beta actin (a*ctβ*) and 40S ribosomal protein S30 (*fau)* as housekeeping genes.

### 2.7 Statistical analysis

All data were analyzed using two-way ANOVA and a Tukey’s honestly significant difference (HSD) as a *post hoc* test, using the Graph Prism 7.0 software (GraphPad Software, Inc., San Diego, CA). A probability level with a *p*-value *<* .05 was used as the minimum to indicate the statistical significance.

## 3 Results

### 3.1 Assessment of cortisol and glucose plasma levels

**FIGURE 1:**
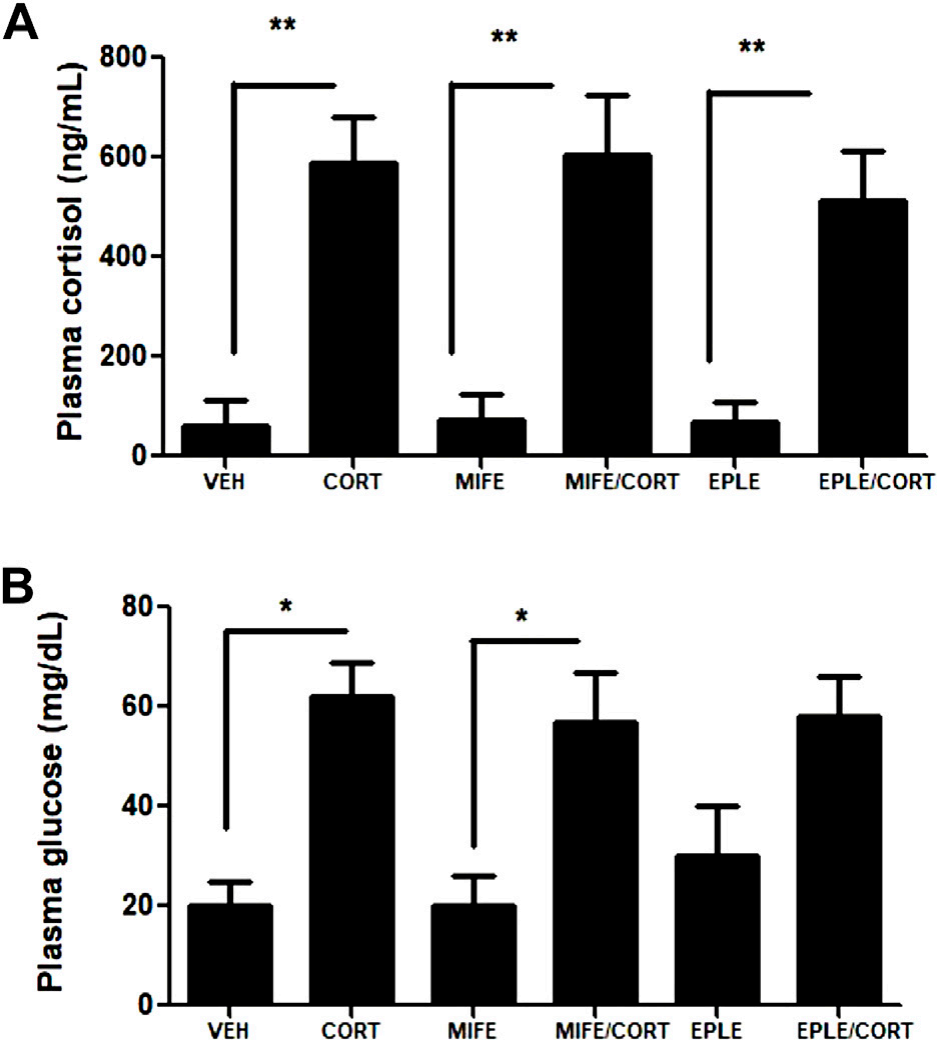
Plasma cortisol and glucose levels of rainbow trout. Cortisol **(A)** and glucose **(B)** levels were measured in the plasma of fish intraperitoneally treated with vehicle (VEH), cortisol (CORT), mifepristone (MIFE), eplerenone (EPLE), mifepristone plus cortisol (MIFE/CORT), eplerenone plus cortisol (EPLE/CORT). The results are expressed as a mean ± SEM, (*n* = 4). The “*” (*p* < .05) and “**” (*p* < .01) represent significant differences compared with the vehicle, mifepristone, eplerenone groups.

Juvenile rainbow trout were intraperitoneally injected with cortisol (10 mg/kg) in the presence or absence of a GR antagonist (mifepristone, 1 mg/kg) or a MR antagonist (eplerenone, 1 mg/kg). To inhibit the endogenous production of cortisol because of the fish manipulation, the individuals were pretreated with the endogenous cortisol inhibitor, metyrapone (1 mg/kg). Additionally, we quantified the basal levels of plasmatic cortisol in a non-manipulated group (33 ± 12 ng/mL), showing no significant differences with respect to the vehicle group (60 ± 53 ng/mL). As expected, plasma cortisol levels increased significantly in the cortisol (590 ± 89 ng/mL), mifepristone/cortisol (605 ± 120 ng/mL), eplerenone/cortisol (531 ± 101 ng/mL) groups compared to the vehicle (60 ± 53 ng/mL) (Figure 1A). There were no significant changes in the levels of cortisol in the plasma of fish treated only with mifepristone (71 ± 55 ng/mL) and eplerenone (68 ± 41 ng/mL) groups compared to vehicle. Similarly, there were a significant increases in plasma glucose levels in the cortisol (62 ± 7 mg/dL) and mifepristone/cortisol (57 ± 10 mg/dL) groups compared to the vehicle (20 ± 5 mg/dL) (Figure 1B). There were no significant changes in the levels of glucose in the plasma of fish treated only with mifepristone (20 ± 6 mg/dL) and eplerenone (31 ± 9 mg/dL) compared to vehicle. In addition, no significant differences were observed in glucose levels when comparing cortisol vs. mifepristone/cortisol and cortisol vs. eplerenone/cortisol treatments.

### 3.2 Transcriptomic responses of rainbow trout skeletal muscle in cortisol vs. mifepristone/cortisol and cortisol vs. eplerenone/cortisol groups

To determine the transcriptional response associated with cortisol and potentially modulated by GR and MR, we sequenced the RNA from the skeletal muscle of fish treated for 3 h with vehicle, cortisol, mifepristone, mifepristone/cortisol, eplerenone, and eplerenone/cortisol. A total of 660, 922, 848 million reads were obtained corresponding to 24 cDNA libraries. After discard adapters and low-quality reads were eliminated, we obtained 618, 153, 281 high-quality reads that were used for further RNA-seq analysis. A total of 433,592,411 high-quality reads (70.14%) were uniquely mapped against the 71,413 CDS of rainbow trout. Principal components analysis of the cDNA libraries of all sampled points are indicated in the Supplementary Figure S1.

To identify differentially expressed transcripts (DETs), samples were subjected to a series of paired comparisons against vehicle group. With cortisol treatment (vehicle vs. cortisol), 266 DETs were identified (255 up and 11 down). The complete list of the differentially expressed transcripts is included in Supplementary Table S2. The 266 differentially expressed transcripts (DETs) were analyzed using the DAVID database and categorized as biological process, molecular function, cellular component, and KEGG pathways. DETs were significantly enriched in a wide variety of biological processes, such as cell-cell adhesion, ion transport, and anion transmembrane transport. Among others relevant biological processes enriched include integrin-mediated signaling pathway, positive regulation of gene expression, regulation of cell proliferation, fatty acid metabolic process, glucose transmembrane transport, proteolysis, and muscle contraction (Table 1). For molecular function and cellular component, the most impacted GO terms were assigned to transmembrane transporter activity and apical plasma membrane, respectively (Supplementary Table S3). KEGG pathway analysis revealed that transcripts associated with pathogenic *Escherichia coli* infection, bile secretion, and metabolism of xenobiotics by cytochrome P450 were differentially expressed (Supplementary Table S3).

**TABLE 1:** Biological process GO terms of differentially expressed transcripts between the vehicle vs. cortisol groups.

GO ID	GO term	Gene count	*p*-value
GO:0098609	cell-cell adhesion	17	1.38E-10
GO:0006811	ion transport	13	2.44E-08
GO:0098656	anion transmembrane transport	6	4.06E-06
GO:0007229	integrin-mediated signaling pathway	8	1.47E-04
GO:0010628	positive regulation of gene expression	15	9.33E-04
GO:0042127	regulation of cell proliferation	7	7.24E-03
GO:0006631	fatty acid metabolic process	5	1.70E-02
GO:1904659	glucose transmembrane transport	3	2.53E-02
GO:0006508	proteolysis	10	2.66E-02
GO:0006936	muscle contraction	4	6.31E-02

We analyzed the participation of glucocorticoid receptor in gene expression under endogenous suppressed cortisol production through the *in vivo* administration of mifepristone. With mifepristone treatment (vehicle vs. mifepristone), 134 DETs were identifies (64 up and 70 down). The complete list of the differentially expressed transcripts is included in Supplementary Table S4. DETs were significantly enriched in biological processes, such as muscle filament sliding, muscle contraction, and ATP metabolic process. Among others relevant biological processes enriched include cardiac muscle contraction, cellular response to heat, intracellular receptor signaling pathway, steroid hormone mediated signaling pathway, positive regulation of transcription from RNA polymerase II promoter, cellular response to oxidative stress, and protein stabilization (Table 2). For molecular function and cellular component, the most impacted GO terms were assigned to microfilament motor activity and sarcomere, respectively (Supplementary Table S5). KEGG pathway analysis revealed that several transcripts associated with hypertrophic cardiomyopathy, dilated cardiomyopathy, PPAR signaling pathway were differentially expressed (Supplementary Table S5).

**TABLE 2:** Biological process GO terms of differentially expressed transcripts between the vehicle vs. mifepristone groups.

GO ID	GO term	Gene count	*p*-value
GO:0030049	muscle filament sliding	9	4.29E-14
GO:0006936	muscle contraction	7	2.93E-07
GO:0046034	ATP metabolic process	5	1.27E-06
GO:0060048	cardiac muscle contraction	5	5.14E-06
GO:0034605	cellular response to heat	3	4.03E-03
GO:0030522	intracellular receptor signaling pathway	3	4.25E-03
GO:0043401	steroid hormone mediated signaling pathway	3	9.35E-03
GO:0045944	positive regulation of transcription from RNA polymerase II promoter	8	1.16E-02
GO:0034599	cellular response to oxidative stress	3	1.17E-02
GO:0050821	protein stabilization	3	4.74E-02

Similarly, we analyzed the participation of mineralocorticoid receptor in gene expression under endogenous suppressed cortisol production through the *in vivo* administration of eplerenone. With eplerenone treatment (vehicle vs. eplerenone), 111 DETs were identified (61 up and 50 down). The complete list of the differentially expressed transcripts is included in Supplementary Table S6. DETs were significantly enriched in biological processes, such as intermediate filament organization, circadian regulation of gene expression, and negative regulation of transcription from RNA polymerase II promoter. Among others relevant biological processes enriched include microtubule cytoskeleton organization, ion transport, extracellular matrix organization, sodium ion transport, fatty acid metabolic process, negative regulation of transcription DNA-templated, positive regulation of transcription from RNA polymerase II promoter (Table 3). For cellular component and molecular function, the most impacted GO terms for differentially expressed transcripts were assigned to neurofilament and structural constituent of cytoskeleton, respectively (Supplementary Table S7). KEGG pathway analysis revealed that several transcripts associated with protein digestion and absorption, ECM-receptor interaction, and amyotrophic lateral sclerosis were differentially expressed (Supplementary Table S7). A Venn diagram analysis revealed that 48 DETs were common between mifepristone and eplerenone treatments (Supplementary Figure S2), mainly associated to intermediate filament organization biological process.

**TABLE 3:** Biological process GO terms of differentially expressed transcripts in common between the vehicle vs. eplerenone groups.

GO ID	GO term	Gene count	*p*-value
GO:0045109	intermediate filament organization	7	1.83E-07
GO:0032922	circadian regulation of gene expression	6	7.30E-06
GO:0000122	negative regulation of transcription from RNA polymerase II promoter	14	6.22E-05
GO:0000226	microtubule cytoskeleton organization	4	1.44E-02
GO:0006811	ion transport	4	1.62E-02
GO:0030198	extracellular matrix organization	4	2.48E-02
GO:0006814	sodium ion transport	3	4.58E-02
GO:0006631	fatty acid metabolic process	3	5.05E-02
GO:0045892	negative regulation of transcription, DNA-templated	6	7.31E-02
GO:0045944	positive regulation of transcription from RNA polymerase II promoter	9	8.39E-02

To understand the effects of administration of a GR antagonist in cortisol-induced gene expression, we determined those DETs that showed a differential expression in the combined treatment of cortisol and mifepristone respect to cortisol administration (cortisol vs. mifepristone/cortisol). The comparative analysis identified 135 DETs (76 up and 59 down). The complete list of the differentially expressed transcripts is included in Supplementary Table S8. DETs were significantly enriched in biological processes, such as inflammatory response, metabolic process, and chloride transmembrane transport. Among others relevant biological processes enriched include oxidation-reduction process, proteolysis, ion transmembrane transport, adaptive immune response, cell proliferation, response to hypoxia, and protein folding (Table 4). In cellular components and molecular function, we found extracellular exosome and chaperone binding as the most impacted, respectively (Supplementary Table S9). KEGG pathway analysis revealed that several transcripts associated with leukocyte transendothelial migration, neutrophil extracellular trap formation, and leishmaniasis were differentially expressed (Supplementary Table S9).

**TABLE 4:** Biological process GO terms of differentially expressed transcripts in common between the cortisol vs. mifepristone/cortisol groups.

GO ID	GO term	Gene count	*p*-value
GO:0006954	inflammatory response	8	6.74E-03
GO:0008152	metabolic process	5	1.69E-02
GO:1902476	chloride transmembrane transport	4	1.71E-02
GO:0055114	oxidation-reduction process	9	2.24E-02
GO:0006508	proteolysis	8	2.70E-02
GO:0034220	ion transmembrane transport	5	3.46E-02
GO:0002250	adaptive immune response	4	5.57E-02
GO:0008283	cell proliferation	6	6.31E-02
GO:0001666	response to hypoxia	4	7.95E-02
GO:0006457	protein folding	4	8.83E-02

Similarly, the comparative analysis of cortisol vs. eplerenone/cortisol, identified 68 DETs (22 up and 46 down). The complete list of the differentially expressed transcripts is included in Supplementary Table S10. We found that the most enriched biological processes were muscle contraction, sarcomere organization, and cardiac muscle contraction. Among others relevant biological processes enriched include skeletal muscle contraction, transition between fast and slow fiber, muscle filament sliding, circadian regulation of gene expression, positive regulation of transcription DNA-templated, regulation of cell cycle, and regulation of neuron differentiation (Table 5). In cellular component and molecular function, sarcomere and actin binding were highly represented, respectively (Supplementary Table S11). In KEGG pathways, hypertrophic cardiomyopathy, dilated cardiomyopathy, and cardiac muscle contraction were overrepresented (Supplementary Table S11). A Venn diagram analysis revealed that only 2 DETs (*zfand5*, *tnni2*) were common between cortisol vs. mifepristone-cortisol and cortisol vs. eplerenone-cortisol (Supplementary Table S3), mainly associated to skeletal system morphogenesis. In all experimental groups no significant differences were detected in the expression of *gr1*, *gr2*, and *mr* (Supplementary Table S12).

**TABLE 5:** Biological process GO terms of differentially expressed transcripts in common between the cortisol vs. eplerenone/cortisol groups.

GO ID	GO term	Gene count	*p*-value
GO:0006936	muscle contraction	8	4.46E-10
GO:0045214	sarcomere organization	5	1.12E-06
GO:0060048	cardiac muscle contraction	5	1.84E-06
GO:0003009	skeletal muscle contraction	4	2.71E-05
GO:0014883	transition between fast and slow fiber	3	1.43E-04
GO:0030049	muscle filament sliding	3	2.17E-04
GO:0032922	circadian regulation of gene expression	3	9.10E-03
GO:0045893	positive regulation of transcription, DNA-templated	6	1.42E-02
GO:0051726	regulation of cell cycle	4	2.59E-02
GO:0045664	regulation of neuron differentiation	3	5.91E-02

### 3.5 Validation of *in silico* data by real-time PCR

**FIGURE 2:**
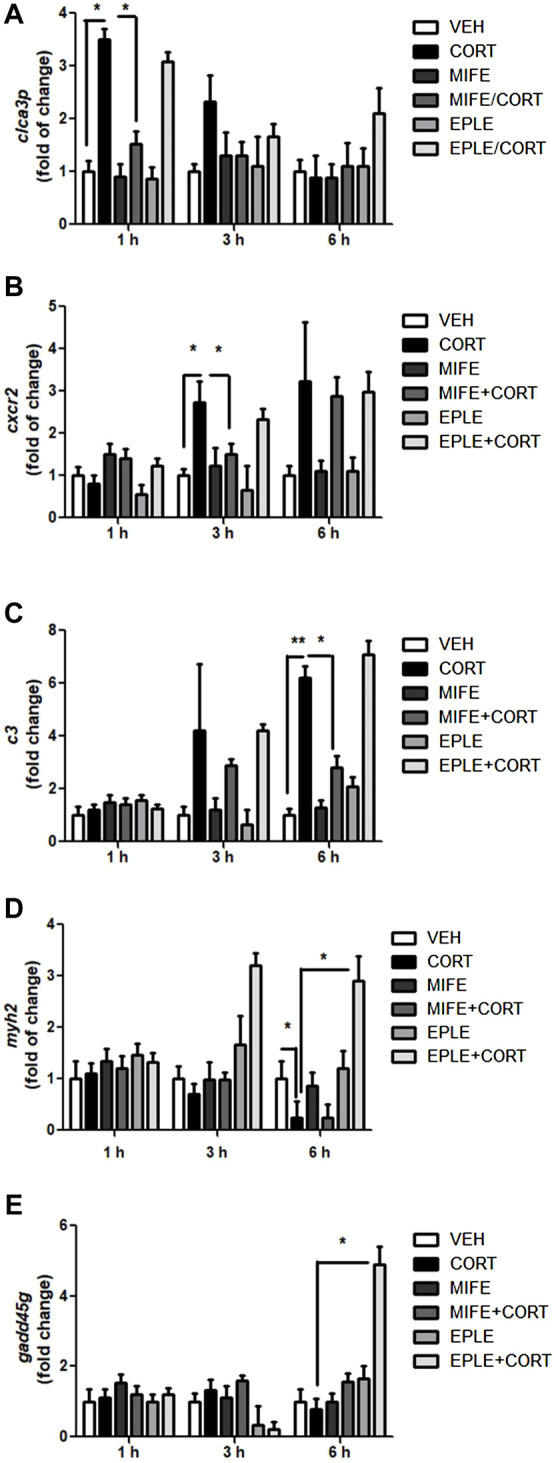
qPCR validation of selected candidate transcripts in rainbow trout myotubes. **(A–E)** *cxcr2*, *c3*, *clca3p*, *myh2*, *gadd45g* expression in rainbow trout myotubes stimulated with cortisol (CORT) in the presence or absence of mifepristone (MIFE) or eplerenone (EPLE) for each indicated times. mRNA levels were analyzed by RT-qPCR and showed as a relative expression normalized against *fau* (40S ribosomal protein S30) as a housekeeping gene. The results are expressed as a mean ± SEM, (*n* = 6). The “*” (*p* < .05) and “**” (*p* < .01) represent significant differences compared with the vehicle, mifepristone, eplerenone groups. Abbreviations: cxcr2: C-X-C chemokine receptor type 2; c3: Complement C3; clca3p: Calcium-activated chloride channel regulator family member 3; myh2: Myosin-2; gadd45g: Growth arrest and DNA damage-inducible protein.

To validate the RNA-seq analysis and the differential participation of GR in contrasting biological processes, we selected a representative gene of inflammatory response (*cxcr2*), proteolysis (*c3*), and ion transmembrane transport (*clca3p*). Similarly, to determine the differential participation of MR, we selected a representative gene of muscle contraction (*myh2*), and regulation of cell cycle (*gadd45g*). The mRNA expression of *cxcr2*, *c3*, *clca3p*, *myh2*, *gadd45g* were monitored in myotube lysates 1, 3, and 6 h following cortisol treatment (500 nM) in the presence or absence of mifepristone (50 nM) or eplerenone (50 nM). At 1 h post-treatment, maximum increases in *clca3p* mRNA expressions were observed (Figure 2A). At 3 h post-treatment, maximum increases in *cxcr2* mRNA expressions were observed (Figure 2B). At 6 h post-treatment, there was a maximum increase in *c3* mRNA expression (Figure 2C). At 6 h post-treatment, there was a maximum decrease in *myh2* mRNA expression (Figure 2D). No significant changes were observed in the expression of *gadd45g* under cortisol treatment (Figure 2E). Pretreatment of skeletal myotubes with the mifepristone significantly inhibited the cortisol-induced up-regulation of *cxcr2*, *c3*, and *clca3p*. Pretreatment of skeletal myotubes with the eplerenone significantly inhibited the cortisol-induced down-regulation of *myh2*. Significant changes in gene expression were observed for *gadd45g* under cortisol treatment in the presence of eplerenone.

## 4 Discussion

In the present study, we determined the potential contribution of glucocorticoid and mineralocorticoid receptors in global transcriptomic response of fish skeletal muscle induced by cortisol. It is important to mention that the control condition of our trials (vehicle group) corresponds to rainbow trout anesthetized with benzocaine and treated with metyrapone, registering a slight (non-significant) increase in plasma cortisol levels respect to non-manipulated individuals. In addition, the intraperitoneal administration of 10 mg/kg of plasmatic cortisol reached higher levels compared to a stressing condition. It has been described that typical cortisol elevations in response to acute stress in rainbow trout tend to range approximately ∼100 ng/mL (López-Patiño et al., 2014) but there are reported notable exceptions in other salmonid species (Maule et al., 1988; Mazik et al., 1991). The protocol implemented in this study has been previously validated by several publications (Aedo et al., 2019a; Aedo et al., 2019c; Aedo et al., 2019b; Aravena-Canales et al., 2021). Similarly, the co-administration of cortisol with mifepristone or eplerenone achieved similar plasma concentrations validating our experimental design. We determined that cortisol administration induced an increase in plasma glucose levels, which was not suppressed by the administration of mifepristone. Although this result is contradictory to that reported in higher vertebrates (Castinetti et al., 2010), we estimate that this difference may be due to the concentration of mifepristone used as well as its route of administration. However, additional experiments will be required to demonstrate this hypothesis. In addition, the administration of mifepristone or eplerenone alone did not induce changes in plasma glucose levels. These results agree with observations obtained in the same species (Vijayan et al., 1994).

To better identify the molecular mechanism mediated by GR or MR in skeletal muscle, we analyzed gene expression through RNA-seq. In general terms, we determined in cortisol vs. mifepristone/cortisol analysis that GR has a potential participation in the expression of transcripts associated with inflammatory response, ATP metabolic process, chloride transmembrane transport, oxidation-reduction process, proteolysis, ion transmembrane transport, adaptive immune response, among others. On the other hand, in cortisol vs. eplerenone/cortisol analysis, MR has a potential participation in the expression of transcripts associated with muscle contraction, sarcomere organization, transition between fast and slow fiber, muscle filament sliding, circadian regulation of gene expression, positive regulation of transcription, regulation of cell cycle. It is important to emphasize that the results obtained indicate potential targets mediated by GR and MR. This is mainly due to the limitation in determining the *in vivo* specificity of mifepristone and eplerenone as GR and MR antagonists, and also because the analyzes of differential expression were circumscribed to paired comparisons (cortisol vs. antagonist/cortisol). However, for future experiments it will be important to consider the incorporation of controls to determine the specificity of both antagonists, as well as to include other differential expression comparative analyzes such as antagonist vs. antagonist/cortisol. Interestingly, the results obtained by this *in silico* approach were validated using primary culture of rainbow trout muscle cells, revealing the regulatory role of GR in inflammatory response, proteolysis, and ion transmembrane transport. MR has a regulatory role in muscle contraction and regulation of cell cycle. These results suggest the differential participation of GR and MR in skeletal muscle biological processes in lower vertebrates, as has been described in mammals (Howard et al., 2022).

As expected, exogenous cortisol modulated 3 hours post administration the differential expression of a reduced number of transcripts. These transcripts were associated with biological processes such as cell-cell adhesion, ion transport, proteolysis, among other processes. These categories of biological processes modulated by cortisol are consistent, with recent studies describing the genomic and non-genomic effects of cortisol on the regulation of gene expression in rainbow trout (*Oncorhynchus mykiss*) and Gilthead Sea Bream (*Sparus aurata*) skeletal muscle (Aedo et al., 2019a; Aedo et al., 2019c; Aedo et al., 2019b; Aedo et al., 2021; Aravena-Canales et al., 2021). Interestingly, in the present study, glucose transport appears as a category of biological processes overrepresented under cortisol treatment. Although increases in plasma glucose after stress events have been widely described in species like Coho salmon (*Oncorhynchus kisutch*) or European eel (*Anguilla*) (Wendelaar Bonga, 1997; Mommsen et al., 1999; Kuo et al., 2013), reports associated with modulation of genes associated to glucose transport mediated by cortisol in vertebrate skeletal muscle are limited (Gray et al., 2006). Among the differentially expressed transcripts associated with glucose transport, we identified in the present study *slc2a11* and *slc5a1*. *slc2a11* encodes for a class II sugar transport facilitator (GLUT11), and *slc5a1* encodes for sodium/glucose cotransporter 1 (SGLT1). Although both transporters have shown increases in their expression under glucocorticoid treatment in mammalian models, there are no antecedents reported in lower vertebrates (Zhao and Keating, 2007). We hypothesize that *slc2a11* and *slc5a1* differential expression contribute to the increase in plasma glucose in fish under stress. These transcripts did not show changes in their differential expression in the presence of mifepristone or eplerenone, suggesting an alternative mechanism in their regulation. Interestingly, it has been described in mammalian myotubes that glucocorticoids inhibit glucose uptake *via* a non-genomic mechanisms (Gong et al., 2016), which suggests a potential non-genomic mechanism in their expression. Taken together, these results confirm the fundamental role of cortisol in glucose homeostasis during the early response to stress. It has also been described that prolonged cortisol secretion induces catabolic-related process, mainly mediated by ubiquitin proteasome (UPS) and autophagy lysosomal (ALS) systems (Aedo et al., 2015; Valenzuela et al., 2018). Consistently, we observed in the present work changes in the expression of transcripts associated with proteolysis.

Among the biological processes modulated by GR, we identified proteolysis and inflammatory response. It is well known that stressful conditions can differentially affect the immune response depending on stressor duration (Rebl and Goldammer, 2018). An acute stressor stimulates the immune response, particularly innate responses, while a chronic stressor suppresses the immune response, enhancing infection susceptibility (Nardocci et al., 2014). We determined that GR modulated the expression of *c3* and *cxcr2*, results that were consistent with *in vitro* validation in rainbow trout myotubes*.* C3 protein plays a central role in the activation of the complement system. The complement is a part of the immune system and consists of multiple components with biological functions such as defense against pathogens and immunomodulation (Zheng et al., 2022). The complement system is an ancient defense mechanism present in invertebrates and vertebrate (Bavia et al., 2022). Concordantly, it has been reported in mammals that glucocorticoid receptor antagonist mifepristone inhibited C3 synthesis induced by dexamethasone or hydrocortisone in human alveolar epithelial cell line (Zach et al., 1993). In fish, it has been determined that Eurasian perch (*Perca fluviatilis*) under repeated emersions stress has a reduced *c3* expression (Douxfils et al., 2014). More recently, in rainbow trout it was determined that the acute stress associated with air exposure stress induces the upregulation of *c3* expression (Khansari et al., 2019). *cxcr2* encodes for receptor for interleukin-8 which is a powerful neutrophil chemotactic factor (Matsushima et al., 2022). In mammals, has been observed that blocking of GR with mifepristone significantly diminished the expression of *cxcr2* in an *in vitro* murine allogeneic skin transplantation model induced by dexamethasone (Liao et al., 2014). In fish, the modulation of *cxcr2* expression is unclear, although there are reports linking the expression of its ligand with GR (Philip et al., 2012).

Other biological processes overrepresented under cortisol treatment mediated by GR is ion transmembrane transport. *clca3p* encodes for a calcium-activated chloride channel regulator family member 3 (Altamura et al., 2020). Although there are no previous reports linking stress or cortisol with changes in the expression of this gene in fish skeletal muscle, the role of cortisol in ion and chloride transport is well-documented. Cortisol has a fundamental participation in the adaptation of teleost to a seawater environment through the upregulation of the expression levels of *nkaa1* (Sodium/potassium-transporting ATPase subunit alpha), *nkcc* (Sodium chloride cotransporter), and *cftr* (Cystic fibrosis transmembrane conductance regulator) in gills (Kiilerich et al., 2007; Kiilerich et al., 2011). Cortisol is also involved in adaptation to fresh water. In the gills of euryhaline teleost, cortisol increases the expression of *nkaa1a*, which participates in ion uptake by chloride cells in fresh water (McCormick et al., 2008). In agreement with our observations, it has been determined that ion transmembrane transport associated to the osmoregulatory role of cortisol is mediated by GR, but not by MR. In Atlantic salmon the administration of mifepristone completely block cortisol-induced expression of *nkaa1a* and *nkaa1b* (McCormick et al., 2008; Tipsmark et al., 2009). Furthermore, it has been determined that translational knockdown of GR inhibited sodium uptake in zebrafish (Kumai et al., 2012).

Among the biological processes modulated by MR, we identified regulation of cell cycle. *gadd45g* encodes for growth arrest and DNA damage-inducible protein gamma (Patel et al., 2022). It was found that aldosterone treatment of human renal epithelial cells resulted in significant up-regulation of *gadd45g*. This up-regulation was inhibited by spironolactone, a MR antagonist (Kellner et al., 2003). Interestingly, it has been determined that in vertebrates *gadd45g* is essential for primary sex determination and testis development (Johnen et al., 2013). Consistently, it has been reported on the role of the MR in gonadal differentiation and fish reproduction. *In vitro* cortisol treatment in testis primary culture of Japanese eels (*Anguilla japonica*) induced DNA replication and improves MR-induced spermatogonia proliferation (Ozaki et al., 2006). It was found that MR is expressed in the testicular tissue of rainbow trout, and MR expression increased close to spermiation (Milla et al., 2008). The relationship between the mineralocorticoid receptor and cell cycle regulation has recently been addressed in a murine myotubes. It has been determined that aldosterone inhibited the process of muscle cell differentiation through increased intracellular oxidative stress, a phenomenon that was blocked in the presence of eplerenone (Lee et al., 2021), suggesting a fundamental role of MR in the modulation of muscle cell proliferation and differentiation.

In the present work, one of the most impacted biological processes under treatment with eplerenone corresponds to skeletal muscle contraction. *myh2* encodes for myosin 2, a fundamental protein for skeletal muscle cytoskeleton organization. Our observations are consistent with results published in human skeletal muscle (Howard et al., 2022). Similar to teleost skeletal muscle, MR is present in various types of mammals muscle groups, as well as in differentiated myoblasts and myotubes (Chadwick et al., 2015). The treatment of human myotubes combined with aldosterone and eplerenone modulated the expression of genes associated with muscle contraction, in particular *myh2* expression (Chadwick et al., 2017a). These findings also agree with recent observations in zebrafish. It has been described that during early development, exogenous glucocorticoid (GC) administration reduces body mass in vertebrates, and this is associated mainly with the glucocorticoid receptor (GR) activation. However, a recently developed work using ubiquitous GR and MR knockout zebrafish (*Danio rerio*) in combination with exogenous cortisol treatment revealed that MR activation favors protein deposition and GR activation stimulates proteolysis, while their combined activation is involved in cortisol-mediated growth suppression (Faught and Vijayan, 2020). The same group described that postnatal triglyceride accumulation is regulated by mineralocorticoid receptor activation under basal and stress conditions. Using MR and GR knockout zebrafish, it was demonstrated that MR activation, is involved in triglyceride accumulation during the larval development post feeding (Faught and Vijayan, 2019). However, lipid and triglyceride metabolism were not categories represented under the eplerenone treatments. In addition, we also determined that the administration of mifepristone and eplerenone alone induce changes in gene expression in rainbow trout skeletal muscle. Among the biological processes modulated by both antagonists are cell adhesion, muscle contraction, and transcription. Interestingly, in a study carried out in human myoblasts, the administration of both antagonists modulated the differential expression of a small number of genes in categories similar to those reported in our analysis (Chadwick et al., 2017b), suggesting conserved functionalities of both receptors between higher and lower vertebrates.

## 5 Conclusion

In the present study, we identified for the first time a set of DETs and biological processes, differentially induced by cortisol and the potentially modulated by GR and MR in rainbow trout skeletal muscle. In cortisol vs. mifepristone/cortisol group, biological processes such as inflammatory response, ion transmembrane transport, proteolysis were overrepresented. In cortisol vs. eplerenone/cortisol group, BP were significantly enriched in muscle contraction, and regulation of cell cycle. These results suggest that both receptors have a differential participation to stress response in teleost skeletal muscle.

## Data Availability

The datasets presented in this study can be found in online repositories. The names of the repository/repositories and accession number(s) can be found below: https://www.ncbi.nlm.nih.gov/bioproject/PRJNA518130.
