# Supplementary figures and images for: Role of glucocorticoid and mineralocorticoid receptors in rainbow trout (Oncorhynchus mykiss) skeletal muscle: A transcriptomic perspective of cortisol action
Source: Front Physiol. 2023 Jan 6;13:1048008. doi: 10.3389/fphys.2022.1048008 (PMC9852899; doi:10.3389/fphys.2022.1048008)

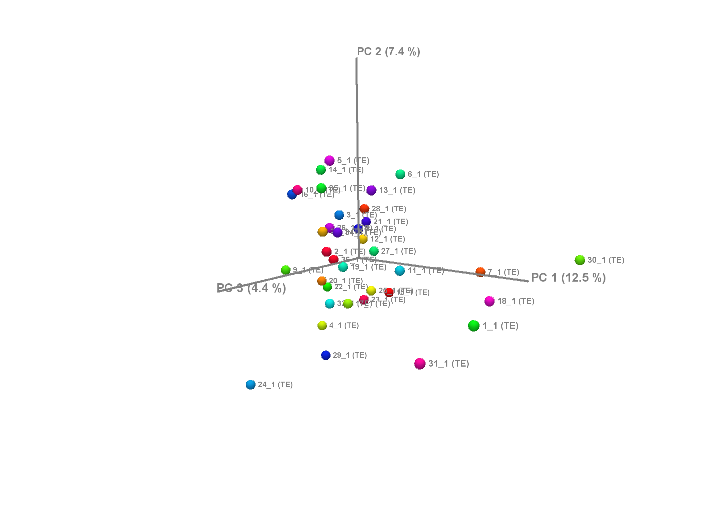

Supplement: Supplementary file 4 [file Image3.TIF]

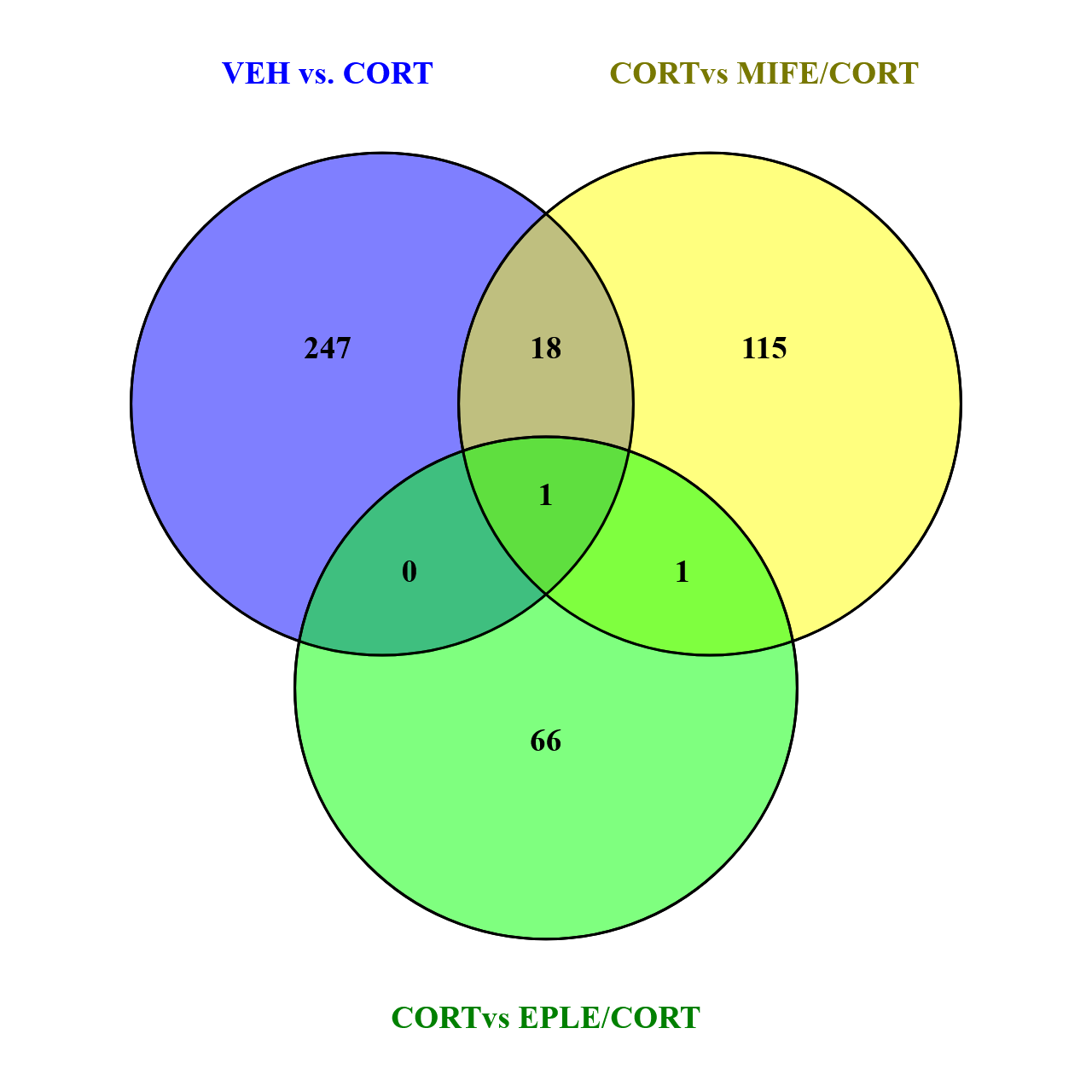

Supplement: Supplementary file 8 [file Image2.PNG]

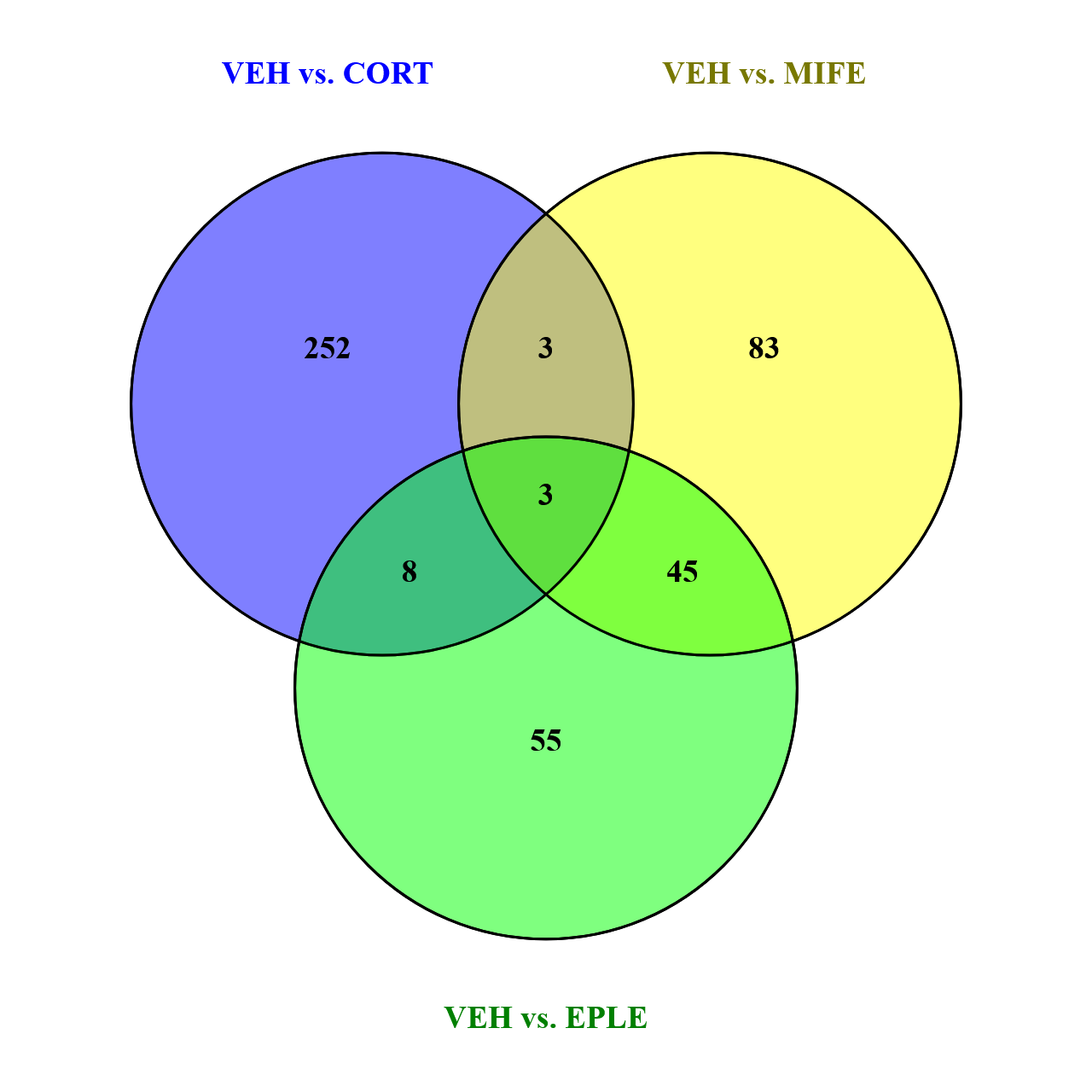

Supplement: Supplementary file 10 [file Image1.PNG]
